# Transfusion or Timing: The Role of Blood Volume in Delayed Cord Clamping During the Cardiovascular Transition at Birth

**DOI:** 10.3389/fped.2019.00405

**Published:** 2019-10-09

**Authors:** Fiona J. Stenning, Stuart B. Hooper, Martin Kluckow, Kelly J. Crossley, Andrew W. Gill, Euan M. Wallace, Arjan B. te Pas, Domenic LaRosa, Graeme R. Polglase

**Affiliations:** ^1^The Ritchie Centre, The Hudson Institute of Medical Research, Monash University, Clayton, VIC, Australia; ^2^Department of Obstetrics and Gynaecology, Monash University, Clayton, VIC, Australia; ^3^Department of Neonatology, Royal North Shore Hospital and Sydney University, St Leonards, NSW, Australia; ^4^Centre for Neonatal Education and Research, The University of Western Australia, Crawley, WA, Australia; ^5^Division of Neonatology, Department of Paediatrics, Leiden University Medical Centre, Leiden, Netherlands

**Keywords:** umbilical cord clamping, delayed cord clamping, preterm birth, newborn infants, resuscitation

## Abstract

Placental transfusion has been thought to be the main benefit of delayed umbilical cord clamping (DCC) in preterm neonates. However, the importance of cardiovascular stability provided by allowing lung aeration prior to cord clamping has recently been highlighted. We aimed to determine the influence of blood volume changes on cardiovascular stability at birth. Preterm lambs (0.85 gestation) were instrumented for measurement of pulmonary, systemic and cerebral blood pressures and flows, systemic oxygen saturation and cerebral oxygenation. Left ventricular output (LVO) was assessed by Doppler Echocardiography. Lambs underwent immediate cord clamping followed by (1) 25 ml/kg infusion of whole blood over (90 s; or 2) withdrawal of 10 ml/kg blood over 90 s. Ventilation was initiated 30 s after volume change (2 min after cord clamping) and was maintained for 30 min. Blood infusion significantly increased pulmonary blood flow (PBF) which maintained systemic cardiac output during the infusion, and increased carotid arterial pressure, flow and heart rate, which remained elevated until after ventilation onset. Upon completion of transfusion PBF rapidly returned to control levels and LVO decreased. Conversely, blood withdrawal decreased PBF and LVO. The cardiovascular changes that accompanied ventilation onset were similar between groups. Providing a blood volume transfusion immediately after umbilical cord clamping maintains PBF and cardiac output during the transfusion, which does not persist beyond the period of the transfusion. Our study implies that an apneic newborn cannot maintain cardiac output through an increase blood volume alone. Importantly, delaying umbilical cord clamping until after breathing/aeration of the lung may be a way to maintain cardiac output throughout delivery at birth.

## Introduction

Delayed umbilical cord clamping (DCC) has been proposed as a mechanism for supporting preterm infants at birth and is now recommended as standard of care for infants not requiring resuscitation. In separate studies, DCC has been shown to improve hemodynamic stability, reduce mortality, decrease the incidence of necrotising enterocolitis and late onset sepsis, reduce the risk of intraventricular hemorrhage (IVH), and increase haematocrit levels in preterm infants after birth (1–3). In a recent meta-analysis DCC in preterm infants has been confirmed to significantly reduced mortality (4). However, the mechanisms underlying the benefits of DCC in preterm infants are not well-understood. Most commentaries attribute the benefits to increased placenta-to-baby blood transfusion. This is despite studies in animals (5) and humans (6, 7) showing that aerating the lung and increasing pulmonary blood flow (PBF) prior to cord clamping (physiological-based cord clamping), maintains cardiac output during transition. As most clinical reports of DCC have not recorded the timing of ventilation onset in relation to cord clamping, until recently the importance of ventilation in stabilizing the cardiovascular transition has not been recognized.

At any moment in time, about 30% of circulating fetal blood volume is contained within the placenta (8). Thus, if cord clamping occurs within 5 s of birth about a third of the total fetal/placental blood volume will remain in the placenta. However, if cord clamping is delayed then the volume of blood retained in the placenta is thought to decrease in a time dependent manner, with the maximal amount of blood (~25 mL/kg) being transfused to the infant within 3 min (9). This concept is consistent with the finding of higher newborn hemoglobin and haematocrit levels at 4 and 24 h after birth and with increased birth weights compared to infants undergoing immediate cord clamping (10). However, it should be noted that the two largest trials on DCC in preterm infants showed that birth weight was not different (APTS) or was reduced after delayed cord clamping (CORD). On the other hand, a meta-analysis comparing “expectant” vs. “active” management of the 3rd stage of labor revealed that active management, which includes immediate cord clamping, is associated with a lower birth weight (11). It is assumed that this reduction in birth weight results from a lack of placental transfusion.

More recently, it has been shown that ventilation (lung aeration) prior to umbilical cord clamping stabilizes cardiovascular function at birth by maintaining cardiac output during the transition to newborn life in preterm lambs (5). Cord clamping prior to ventilation onset rapidly increases arterial pressure, due to removal of the low resistance placental circulation, and reduces cardiac output due to a reduction in preload caused by the loss of umbilical venous return. In contrast, establishing ventilation prior to cord clamping increases PBF, which can immediately replace umbilical venous return upon cord clamping. As a result, blood return to the left heart is maintained which, in turn, sustains cardiac output thereby maintaining arterial and cerebral oxygenation (12). Similarly, two recent human studies have shown that when cord clamping is delayed until after infants (preterm and near term) commence breathing, the infants have a higher heart rate at 1 and 2 min after birth than would be predicted from nomograms of “healthy” infants (6, 7). It is important to note that these nomograms were generated from infants who received immediate cord clamping, which is likely to be the cause of the low heart rate immediately after birth (13). While the maintenance of cardiac output (by maintaining ventricular preload) is increasingly being recognized as the primary benefit of DCC, the role of increase blood volume (placental transfusion) on cardiac output during DCC is unknown. Similarly, it is not known whether a reduction in blood volume due to the absence of placental transfusion (or a reverse transfusion) could underlie poor cardiac output following immediate cord clamping. We sought to resolve those unanswered questions.

In this study we aimed to isolate the effects of blood volume on the cardiovascular transition at birth. We investigated the effect of independently reducing and increasing blood volume after immediate cord clamping in preterm lambs. We hypothesized that the transfusion of whole blood would sustain cardiac output during the period between cord clamping and ventilation onset while reducing blood volume would reduce cardiac output.

## Methods

### Experimental Protocol

All experimental procedures were performed in accordance with the National Health and Medical Research Council (NHMRC) Code of Practice for the Care and Use of Animals for Scientific Purposes and were approved by the Monash Medical Center A Ethics Committee at Monash University.

Pregnant ewes (*Ovis aries*, Border-Leicester) bred by the Monash University Research Platform underwent acute surgery at 125 ± 1 (SD) days gestation (term is ~147). Anesthesia was induced using an intravenous bolus of 5% sodium thiopentone (Pentothal; 1 g in 20 mL) and following intubation was maintained with inhaled isoflurane (1.5–3%) in O_2_ and air. Preterm lambs were exposed by hysterotomy and 20-gauge polyvinyl catheters filled with heparinised saline were inserted into the left carotid artery, jugular vein and a femoral artery for measurement of blood pressure. Ultrasonic flow probes (Transonic Systems; Ithaca, NY, USA) were placed around the right carotid artery and the left main pulmonary artery. The trachea was intubated with a 4.5 mm cuffed endotracheal tube and the tube was clamped to prevent lung liquid loss. The fetal forehead was shaved and a near infrared spectroscopy (NIRS) sensor (Foresight CASMED, Branford CT) attached for cerebral oxygenation recording. A pulse oximetry probe (Massimo) was attached to the forelimb. Baseline recordings were taken before lung liquid was passively drained and the umbilical cord was clamped and cut.

Upon umbilical cord clamping, lambs were randomized into 2 groups: (1) lambs received a 25 mL/kg bolus of fetal blood over 90 s (VOL_IN_); or 2) 10 mL/kg of blood was withdrawn over 90 s (VOL_OUT_). The decision to transfuse 25 mL/kg is based findings that ~25 mL/kg of blood is transfused during DCC (14), while withdrawing blood was undertaken to understand what reducing blood volume can have on the cardiovascular transition at birth. Blood was collected from the umbilical vein of pregnant ewes immediately after cord clamping of preterm lambs being delivered for other studies being undertaken concurrently in the research facility. Blood was stored in heparinised syringes at 38 degrees in a hot water bath for a maximum of 60 min prior to infusion. Two minutes after umbilical cord clamping an initial sustained inflation (35 cmH_2_O) was given to all lambs for 30 s by a Neopuff (Fisher & Paykel Healthcare, Panmure, Auckland, New Zealand) before positive pressure ventilation (Dräeger Babylog 8000+ ventilator, Dräeger, Lübeck, Germany) was initiated. Peak inflation pressure (PIP) was set at 35 cm H_2_O, peak end-expiratory pressure (PEEP) at 5 cm H_2_O, tidal volume (Vt) at 7.0 mL/kg with volume guarantee, fraction of inspired oxygen (FiO_2_) at 21% and respiratory rate at 60 breaths per minute (BPM). FiO_2_, respiratory rate and PIP were adjusted to maintain a target arterial oxygen saturation (SpO_2_) of 90–95% and partial pressure of carbon dioxide (PaCO_2_) of 45–55 mmHg. An infusion of alfaxane (5–10 mg/kg/h; Jurox, East Tamaki, Auckland, New Zealand) in 5% dextrose was commenced to maintain analgesia and anesthesia for the duration of the experiment.

Echocardiogram examinations were performed prior to cord clamping and continuously throughout the first 3 min, with samples recorded at 30, 60, 90, 120, 150, 180, 300, 900, and 1,800 s after cord clamping for analysis (Philips CX50 Compact Extreme, 12-4MHz Neonatal Cardiac Transducer, Philips Healthcare, New South Wales, Australia). The left ventricular output (LVO) was visualized from a five chamber (four chamber plus aorta) apical view and the outflow velocity recorded as a pulse wave Doppler trace with the sample gate in the center of the aorta immediately above the aortic valve. Velocity time integral (VTI) was averaged over at least 5 beats at each time point. The aortic valve annulus diameter was measured from the same view, using an average of at least 3 views. It was assumed that this measurement remained constant over the duration of the study. Instantaneous heart rate was derived from the pulse wave aortic velocity trace. LVO was calculated as VTI • π • (Aortic Diameter^2^/4) • heart rate, standardized by birthweight.

All lambs were ventilated for 30 min after umbilical cord clamping and were euthanased using sodium pentobarbitone (100 mg/kg IV, Lethobarb, Virbac, Australia Pty Ltd) while under general anesthesia. Ewes were also euthanased with sodium pentobarbitone (100 mg/kg IV) following delivery of the lamb(s).

### Statistics

Baseline fetal data were analyzed using a Student's *t*-test. Descriptive physiological data is presented as mean and standard deviation.

Blood gas measurements were taken from the right carotid arterial catheter prior to cord clamping, 15 s prior to ventilation, and at 5-min intervals from ventilation until the end of the experiment. The data collected were analyzed using two-way repeated measures of ANOVA with *post hoc* analysis (Holm–Sidak; Sigmastat v3.0, SPSS Inc.).

Physiological parameters were recorded using LabChart (ADInstruments, NSW, Australia) and analyzed offline. Ten heart beat averages were taken every 30 s from immediately prior to, during and following cord clamping, volume changes, and ventilation onset. Physiological data were analyzed using two-way repeated measures of ANOVA with *post hoc* analysis (Holm–Sidak). Statistical significance was accepted as *p* < 0.05.

## Results

### Fetal Characteristics

Fetal body weight, sex, and blood gas parameters were all similar prior to delivery (Table 1).

**Table 1:** Fetal characteristics.

	**VOL_**IN**_**	**VOL_**OUT**_**	***p*-value**
n (males)	6 (2)	6 (3)	0.699
Twin (first twin)	6 (3)	6 (3)	1.000
Weight (kg)	3.01 ± 0.2	3.07 ± 0.2	0.787
pH	7.27 ± 0.01	7.27 ± 0.02	0.863
PaO_2_ (mmHg)	28.8 ± 1.4	26.84 ± 2.8	0.608
PaCO_2_ (mmHg)	56.0 ± 2.4	55.61 ± 3.7	0.892
SaO_2_ (%)	75.2 ± 3.7	68.3 ± 7.7	0.261
Hb (g.dl^−1^)	11.5 ± 0.6	10.4 ± 0.4	0.505

*Baseline fetal characteristics of sex, birth order, weight, and blood gas values of pH, partial pressure of arterial (Pa) oxygen (O_2_), carbon dioxide (PaCO_2_), arterial oxygen saturation (SaO_2_), and hemoglobin (Hb) for VOL_IN_ and VOL_OUT_ lambs presented as mean ± SEM*.

### Arterial Blood Gas Parameters

**Figure 1:**
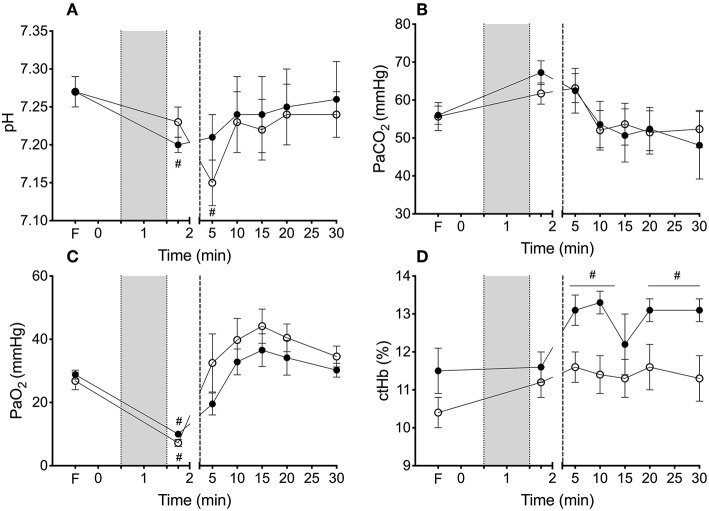
Arterial blood gas parameters during cord clamping, volume transfusion and ventilation. Arterial blood gas values of **(A)** pH, **(B)** partial pressure of arterial (Pa) oxygen (O_2_), **(C)** carbon dioxide (PaCO_2_), and **(D)** concentration of hemoglobin (ctHb) for VOL_IN_ and VOL_OUT_ lambs. ^#^Indicate difference at that time from fetal value (*P* < 0.05). *Indicates significant difference between VOL_IN_ vs. VOL_OUT_ (*p* < 0.05). ^#^Indicates significant difference from fetal value (*p* < 0.05).

Arterial blood gas parameters are outlined in Figure 1. Fetal arterial blood pH, partial pressure of carbon dioxide (PaCO_2_) and partial pressure of oxygen (PaO_2_) were not different between groups throughout the study. Hemoglobin concentration appeared higher in the VOL_IN_ lambs than VOL_OUT_ lambs from 10 min until the end of the study however this did not reach significance. Core body temperature was not different between groups during this study (mean ± SD; VOL_IN_ 36.7 ± 0.4°C vs. VOL_OUT_ 37.2 ± 0.4°C).

## Volume Change

### The Effect of Volume Change on Physiological Parameters

**Figure 2:**
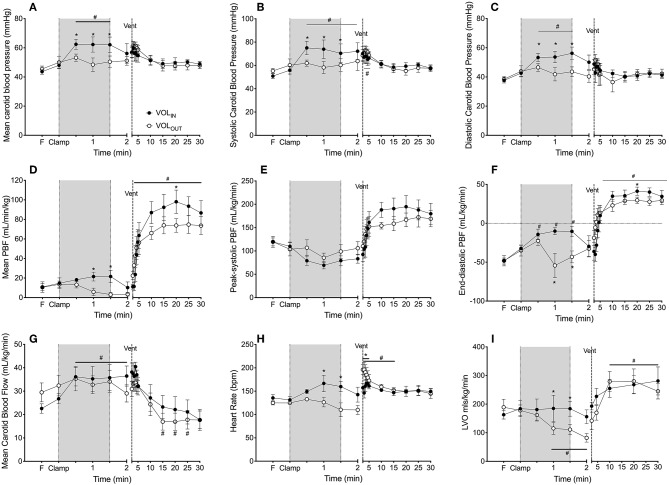
Effect of volume and ventilation on physiological parameters. **(A)** Mean, **(B)** systolic and **(C)** diastolic blood pressure, **(D)** mean, **(E)** peak-systolic and **(F)** end-diastolic pulmonary blood flow percentage change from pre-cord clamping level (PBF), **(G)** carotid arterial flow percentage change from pre-cord clamping level (CaF), **(C)** mean carotid arterial blood flow, **(H)** heart rate and **(I)** left ventricular output (LVO) measured in VOL_IN_ (closed circles) and VOL_OUT_ (open circles) preterm lambs immediately prior to (Fetal) and after umbilical cord clamping (clamp), during volume change (shaded area; infusion or withdrawal) and after ventilation onset (dashed line: vent). Lambs were ventilated for 30 min. *Indicates significant difference VOL_IN_ vs. VOL_OUT_ (*p* < 0.05). ^#^Indicates significant difference from fetal value (*p* < 0.05).

Mean carotid arterial pressure (Figure 2A), systolic carotid arterial pressure (Figure 2B) and diastolic P_CA_ (Figure 2C) increased (by 14.7 ± 2.8, 10.9 ± 2.1, and 19.8 ± 4.6 mmHg, respectively) in VOL_IN_ lambs within 30 s of the infusion, and were significantly higher in the VOL_IN_ group compared to the VOL_OUT_ group from 30 to 90 s after cord clamping. Blood pressures were similar between groups 30 s after blood transfusion or withdrawal had been completed (at 120 s).

Mean PBF was maintained in VOL_IN_ lambs during blood transfusion while mean PBF decreased significantly in the VOL_OUT_ group during blood withdrawal and was significantly lower than the VOL_IN_ group 60 s after cord clamping. After completion of the transfusion, PBF decreased in VOL_IN_ lambs such that it was no longer different to VOL_OUT_ lambs 120 s after cord clamping. Peak systolic PBF was not different between groups (Figure 2E). End-diastolic PBF significantly increased from fetal values in both groups by 30 s, while end-diastolic PBF subsequently reduce in VOL_OUT_ lambs during blood withdrawal at 60 and 90 s (Figure 2F). By 120 s end-diastolic PBF was again not different between groups.

Carotid arterial flow was not different between groups prior to ventilation onset. Carotid arterial flow significantly increased from the fetal value in VOL_IN_ lambs from 30 s and remained elevated until after ventilation onset. CaF did not significantly change in the VOL_OUT_ group during the time between cord clamping and ventilation onset (Figure 2G).

Heart rate (HR) was significantly increased in VOL_IN_ lambs during the blood transfusion and was significantly higher than VOL_OUT_ lambs at 60 and 90 s (Figure 2H).

### The Effect of Volume Change on Cardiac Output

Left ventricular output (LVO) was not different between VOL_IN_ and VOL_OUT_ lambs prior to cord clamping (Figure 2I). LVO increased by ~20 ml/min/kg during transfusion in VOL_IN_ lambs but fell (by 28 ml/kg/min) back to fetal levels upon cessation of the transfusion. LVO decreased significantly in VOL_OUT_ lambs following cord clamping and was significantly lower than VOL_IN_ lambs from 60 to 120 s.

Left ventricular outflow tract VTI decreased significantly in the VOL_OUT_ lambs at 60 and 120 s after cord clamping when compared to fetal levels. VTI remained stable in VOL_IN_ lambs during this time. VTI was not significantly different groups at any time point (*p* = 0.066) (Data not shown).

### The Effect of Volume Change on Arterial Oxygen Saturation (SpO_2_) and Cerebral (SctO_2_) Oxygenation

**Figure 3:**
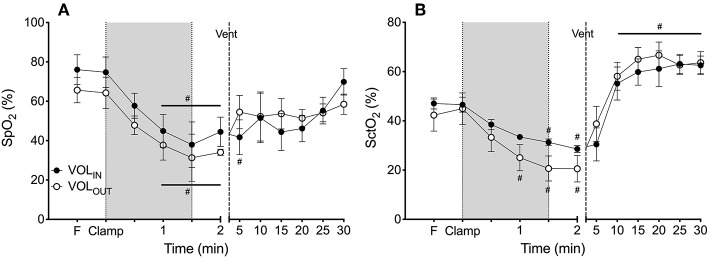
Arterial and cerebral oxygenation. **(A)** Mean systemic arterial oxygen saturation (SpO_2_) and **(B)** mean cerebral tissue oxygenation (SctO_2_) measured in VOL_IN_ (closed circles) and VOL_OUT_ (open circles) preterm lambs throughout the study. ^#^ indicates significant difference from fetal value (*p* < 0.05).

SpO_2_ decreased rapidly after umbilical cord clamping in both the VOL_IN_ and VOL_OUT_ groups (by 35 and 43%, respectively) and was significantly lower than fetal values from 60 s (Figure 3A). No difference was observed between the groups. Similarly, SctO_2_ decreased rapidly after umbilical cord clamping in both the VOL_IN_ and VOL_OUT_ groups (by 49 and 48%, respectively) and was significantly lower than fetal values by 60 s (VOL_OUT_) and 90 s (VOL_OUT_). There was no difference between groups at any time point (Figure 3B).

## Ventilation Onset

Ventilator parameters are presented in Table 2. None of the ventilation or oxygenation parameters were different between groups at any time point.

**Table 2:** Ventilation parameters.

**Time (min)**						
**Blood gas**	**Group**	**5**	**10**	**15**	**20**	**30**
FiO_2_ (%)	VOL_IN_	0.48 ± 0.08	0.46 ± 0.08	0.52 ± 0.08	0.51 ± 0.08	0.55 ± 0.07
	VOL_OUT_	0.57 ± 0.06	0.57 ± 0.06	0.59 ± 0.09	0.59 ± 0.09	0.49 ± 0.06
PIP (cmH_2_O)	VOL_IN_	44.9 ± 1.8	41.6 ± 1.7	41.0 ± 1.4	41.0 ± 1.3	40.3 ± 1.3
	VOL_OUT_	42.3 ± 1.9	42.4 ± 1.7	42.1 ± 1.7	42.6 ± 1.9	42 ± 1.9
Paw (cmH_2_O)	VOL_IN_	20.5 ± 0.8	19.2 ± 0.6	18.5 ± 0.8	18.5 ± 0.7	18.0 ± 0.8
	VOL_OUT_	19.4 ± 0.8	19.4 ± 0.9	19.5 ± 0.8	19.7 ± 0.8	19.5 ± 0.8
OI	VOL_IN_	16.3 ± 2.5	16.7 ± 2.5	19.3 ± 1.9	18.4 ± 2.5	28.3 ± 8.3
	VOL_OUT_	15.6 ± 2.0	22.5 ± 4.3	24.0 ± 6.5	24.6 ± 5.6	18.4 ± 1.9
AaDO_2_	VOL_IN_	257.7 ± 56.8	236.6 ± 52.6	278.8 ± 48.8	272.2 ± 51.4	313.4 ± 40.8
	VOL_OUT_	258.2 ± 40.6	307.7 ± 49.0	316.9 ± 63.5	325.0 ± 61.7	258.4 ± 36.7

*FiO2, Fraction of inspired oxygen; PIP, peak inflation pressure; Paw, mean airway pressure; OI, oxygenation index and alveolar-arterial difference in oxygen (AaDO_2_) for VOL_IN_ and VOL_OUT_ lambs. There are no significant differences in any of the variables between groups*.

### The Effect of Ventilation on Physiological Parameters

Mean, systolic and diastolic P_CA_s were not different between VOL_IN_ and VOL_OUT_ lambs at any time point after ventilation onset, nor did they alter over time (Figures 2A–C).

Ventilation caused a rapid and significant increase in mean, peak-systolic, and end-diastolic PBF in both groups from 30 s after ventilation onset (Figures 2D–F). Mean PBF was higher in VOL_IN_ lambs than VOL_OUT_ lambs from 10 min, reaching significance at 20 min. Peak-systolic and end-diastolic PBF were not different between groups.

CaF decreased significantly from 15 min after ventilation onset in both groups (Figure 2G) as oxygenation improved, but was not different between VOL_IN_ and VOL_OUT_ groups at any time point after ventilation onset.

### The Effect of Ventilation on Cardiac Function

HR was not significantly different between groups at the onset of ventilation onset (i.e., at 2 min; Figure 2H). However, HR significantly increased in the VOL_IN_ lambs from 111.58 ± 11.19 BPM at ventilation onset to 198.75 ± 12.26 BPM at 30 s after ventilation onset and remained higher than VOL_OUT_ lambs for the first 90 s of ventilation (Figure 2H). HR was not different thereafter.

Within 30 s of ventilation onset, LVO was restored to fetal values in VOL_OUT_ lambs and was subsequently not different to VOL_IN_ lambs from 2 min after ventilation onset till the end of the study (Figure 2I).

### The Effect of Ventilation on Arterial Oxygen Saturation (SpO_2_) and Cerebral (SctO_2_) Oxygenation

Ventilation increased SpO_2_ and SctO_2_ similarly in both groups, with no differences between groups detected (Figure 3).

## Discussion

The critical aspect of a smooth cardiovascular transition at birth is the maintenance of preload and cardiac output throughout delivery. While attached to the placental circulation, left ventricular preload is predominantly derived from the umbilical circulation which flows through the ductus venosus and foramen ovale to enter the left ventricle (15, 16). Our study demonstrated that increasing blood volume alone is not able to maintain preload to the left ventricle throughout transition. The only way this can be sustained is through aeration of the lung which enables PBF to increase and become the main source of left ventricular preload. Thus, aeration of the lung, and not increasing blood volume, is the most important aspect for maintaining left ventricular preload, and thus cardiac output, in preterm neonates.

It has been proposed that ~25 mL/kg of blood is transfused from the placenta into the newborn infant during the first three 3 min after birth if the cord remains unclamped (9, 14, 17). Until recently, it was thought that this volume load was responsible for the major benefits associated with DCC after birth. However, most studies investigating DCC have purposefully excluded infants requiring any ventilation assistance after birth. As such, the studies were restricted to healthy infants who commenced breathing immediately at birth and who successfully transitioned to extra-uterine life without assistance. To separate the roles of volume transfusion from that of ventilation onset in maintaining cardiac output after birth, we transfused 25 mL/kg of blood into the jugular vein during the 90 s following cord clamping and then waited 30 s before ventilating the lambs. This allowed us to investigate the physiological effects of volume transfusion separate to the effects of ventilation during the period immediately following birth. We compared this to the withdrawal of blood prior to ventilation, simulating reduced fetal blood volume at transition. Blood transfusion of 25 mL/kg caused a rapid increase in arterial pressure, heart rate and PBF within 30 s, maintaining cardiac output for the duration of the transfusion. However, upon cessation of the transfusion PBF rapidly fell back to control (fetal) levels with an associated fall in heart rate, and LVO reflecting failure to maintain cardiac output. Conversely, withdrawal of blood caused a reduction in LVO in the period between cord clamping and ventilation onset. Ventilation restored PBF, HR, and LVO in VOL_IN_ lambs with no major differences observed between the two transfusion groups after 5 min. These observations demonstrate that the maintenance of LVO during DCC is the most important in aspect in maintaining cardiovascular stability during the transition at birth, and that increasing PBF via lung aeration, and not volume transfusion, is the main determinant of maintaining cardiac output. In short, ensuring a smooth transition at birth is to ensure that the newborn's lungs are aerated before the cord is clamped. We contend that the additional volume transfusion afforded the baby during DCC to ensure lung aeration is simply an epiphenomenon and plays little, if any, role in a healthy transition.

### Haemodynamic Effects of Transfusion

In preterm lambs prior to ventilation onset, immediate cord clamping causes a reduction in cardiac output by up to 50% due to the loss in umbilical venous return and the subsequent reduction in left ventricular preload (18). We found that the transfusion of blood was able to compensate for this and so maintain LVO after cord clamping, but only for the duration of the transfusion. Once the transfusion ended, PBF (and so pre-load), decreased again leading to a decrease in LVO. However, we need to acknowledge that we gave the transfusion via the jugular vein which drains into the super vena cava (SVC). In fetal sheep, the majority of blood returning to the heart via the superior vena cava is thought to enter the right atrium with little traversing the foramen ovale into the left atrium (15). However, following cord clamping (and before ventilation onset) the fate of SVC blood is more complex and essentially unknown. As such, the maintenance of LVO during the blood transfusion could have resulted from additional blood entering the right atrium from the SVC. This would have led to an increase in right ventricular preload and output which, when combined with an increase in systemic vascular resistance, increased PBF and pulmonary venous return. Alternatively, the proportion of SVC derived blood passing through the foramen ovale directly into the left atrium may have increased due to a simultaneous increase in SVC flow and decrease in IVC flow secondary to a loss of umbilical venous return. In this instance, if we gave an infusion by the umbilical vein we may not have observed an increase in PBF. Irrespective, it is clear that the infusion of blood was able to transiently maintain left ventricular preload which, combined with the increase in HR, maintained cardiac output following cord clamping. However, LVO was only sustained during the infusion. Once the transfusion ended PBF decreased rapidly leading to a fall in LVO and an unstable transition.

At birth the huge reduction in pulmonary vascular resistance (PVR) and increase in PBF is a direct consequence of lung aeration (19). The reduction in PVR is so large that the pulmonary circulation can almost immediately accommodate the entire output of the right ventricle, while also reducing pulmonary arterial pressure (17). As a result, the pressure gradient across the ductus arteriosus reverses such that systemic arterial pressure becomes greater than pulmonary arterial pressure. This causes the direction of blood flow across the ductus arteriosus to reverse and, as a result, LVO contributes significantly to high PBF levels immediately after birth (17). It has been hypothesized that the increase in PBF after birth increases pulmonary blood volume by drawing blood into the dilated pulmonary vasculature, thereby accounting for the need of the newborn to increase blood volume during DCC (20). However, the increase in pulmonary blood volume associated with the increased PBF at birth is only 2–3 mL/kg (21). This is explained by the fact that when blood vessels dilate, the volume contained within them increases with the radius squared but the resistance decreases with the radius to the 4th power. This means that the large reduction in PVR at birth occurs with only small changes to the radius of the blood vessels, and thus only a small increase in pulmonary blood volume relative to total blood volume.

The increase in PBF we observed in response to the blood volume infusion, that was prior to lung aeration, was most likely due to a simultaneous increase in right ventricular output in response to preload and the significant increase in systemic arterial resistance caused by cord clamping. Indeed, the increase in PBF was primarily associated with a significant reduction in retrograde PBF during diastole, reducing from −32 ± 4 ml/kg/min at the time of umbilical cord clamping to −10 ± 5 ml/kg/min at the end of the transfusion. This indicates a marked reduction in right to left blood flow through the ductus arteriosus during diastole, likely caused by a rapid increase in blood pressure that reduced the pressure gradient between the pulmonary and systemic circulations [Figure 2 and (5)].

The increase in systemic vascular resistance caused by umbilical cord clamping is a direct result of removing the low resistance placental circulation that is connected in parallel across the lower body. This sudden increase in vascular resistance causes a rapid increase in blood pressure and carotid blood flow that is observed with remarkable consistency across animals (5, 22). However, when cord clamping occurred after reduced blood volume, carotid arterial pressures and flows remained unchanged. It is likely that withdrawing blood volume decreases systemic vascular resistance thus prevented the sudden rise in blood pressure associated with cord clamping.

While transfusion of whole blood prevented the fall in cardiac output associated with cord clamping prior to breathing onset (5), it did not prevent the rapid fall in SctO_2_ and SpO_2_ that occurred immediately following cord clamping and throughout the infusion. The fetal blood administered to the lambs was collected from the umbilical vein of preterm lambs delivered prior to the experiment and was stored for use within 12 h. Unfortunately, blood gas levels were not measured prior to administering blood to the lambs and it is possible that the blood transfused had a lower oxygen content than fresh umbilical venous blood. As such, it is possible that the reduction in SpO_2_ and SctO_2_ in VOL_IN_ lambs may have been prevented during the volume transfusion if the transfused blood had a higher oxygen concentration. However, the rate of infusion (16.6 mL/kg/min) is much lower (by a factor of ~20-fold) than umbilical venous flow and so this would seem unlikely. Similar reductions in SctO_2_ and SpO_2_ has been described previously by Polglase et al. during the period between cord clamping and ventilation onset (12), which was mitigated by delaying cord clamping until after ventilation onset (12). Indeed, a study by Dawson et al. to define the normal reference range for arterial oxygen saturation in the first 10 min of life found that half of infants had an oxygen saturation less than fetal levels at the first minute of life. These reference ranges were based on infants that were born at term, underwent ICC and did not require resuscitation (13). In comparison, studies have shown that the median SpO_2_ is higher in the first minutes after birth in infants that received delayed cord clamping than would be expected from the defined reference range group at 1 min of life (6, 23). Together, these findings highlight the importance of aeration of the lung prior to cord clamping for not only cardiovascular stability but also preventing acute hypoxia at delivery.

### Ventilatory Effects of Transfusion

Aeration of the lungs is the trigger for the cardiopulmonary transition. It allows for a reduction in PVR, an increase in PBF, and provides a low resistance circulation to replace the placenta as the site of gas exchange. Ventilation caused a rapid and significant increase in PBF in both groups. However, in the current study VOL_IN_ lambs maintained a higher mean, peak-systolic and end-diastolic PBF than VOL_OUT_ lambs for the remainder of the study. Despite the higher PBF, cardiac output was not different between groups, indicating that no short-term hemodynamic benefits were observed with changes in blood volume.

Upon ventilation onset, carotid arterial pressure increased by ~9.5 mmHg within the first 60 s in the VOL_OUT_ group compared to ~4.9 mmHg in the VOL_IN_ group. The increase in arterial pressure in the VOL_OUT_ group coincided with a rapid increase in heart rate which increased by ~15 bpm within the first 30 s in the VOL_OUT_ group compared to ~89 bpm in the VOL_IN_ group. The rapid increase in blood pressure and HR likely resulted in response to a lower circulating blood volume, and therefore a reduced venous return, in order to restore cardiac output. The large fluctuations in blood pressure in the VOL_OUT_ were greater than that observed in immediate cord clamping lambs in the Bhatt study (22). Rapid changes in cerebral blood pressure and flow may result in cerebral injury due to the pressure passivity in the preterm circulation (24, 25). A higher newborn blood volume may reduce the degree of blood pressure fluctuations upon ventilation onset in an apnoeic infant, reducing the risk of hypoperfusion and reperfusion injury such as intraventricular hemorrhage (IVH) (26), but not nearly to the same degree that aeration of the lung prior to cord clamping does (22).

Ventilation allowed for a rapid increase in SpO_2_ and SctO_2_ in both groups. The rapid reduction in oxygenation following cord clamping and during the volume change, followed by a rapid increase immediately after ventilation onset was similar to that reported in preterm lambs who underwent immediate cord clamping followed by ventilation (12). This highlights the requirement for adequate ventilation prior to cord clamping in order to prevent hypoxia in the immediate postnatal period.

DCC has been shown to result in significantly higher hemoglobin at 6 h after birth and reduced incidence of anemia (27–29). Similarly, blood transfusion in our study resulted in a higher hemoglobin level at 30 min of life which further supports the role of placental transfusion in the benefits of DCC. Interestingly, while a significant increase in hemoglobin concentration was found following volume transfusion, hemoglobin (Hb) also increased in lambs whom had 10 ml/kg was withdrawn. This intriguing finding suggests that increasing Hb is normal in the first hours after birth in newborns. This most likely occurs due to a shift of water from the vascular compartment (plasma) into multiple extravascular compartments, including loss as urine, that causes increased Hb concentration. Given our finding, it is important to consider the infant's hydration status if Hb is to be used as an indicator of placental transfusion.

There are a number of limitations of this study. We conducted the study in anesthetized lambs in which spontaneous breathing is depressed. This was intentional so we could differentiate the effect of volume and lung aeration on cardiac output. However, the effect of this intervention on spontaneously breathing ewes/lambs is not known so caution should be made directly translating to the clinical paradigm. Our study also used higher ventilator pressures than are routinely used in preterm infants. PIP was set at 35 cm H_2_O compared to the recommended 20–25 cm H_2_O recommended to be used for the resuscitation of preterm infants (30). These pressures are required given that the ewes in this study did not receive antenatal corticosteroids. We have previously shown that antenatal betamethasone increases fetal PBF prior to delivery (31) which would likely increase the amount of preload provided by the pulmonary circulation, which in turn may alter cardiac output upon UCC. However, the increase in preload by antenatal corticosteroids is not nearly as substantial as that which occurs upon aeration of the lung, and they do not increase postnatal PBF thus would unlikely alter the findings in this study (31). But this warrants further investigation. We did not give surfactant to the lambs during the study. While early surfactant is becoming more routine in some units, the time-line of the study (30 min) prevented us from using it, and surfactant would unlikely be given prior to umbilical cord clamping and initiation of respiratory support which was the focus time-period of this study.

## Conclusion

Our studies show that blood volume change in the period between cord clamping and ventilation does affect cardiac output. The transfusion of fetal blood at the time of cord clamping stabilized cardiac output by increasing PBF, but this benefit was lost as soon as transfusion ceased. This indicates blood volume alone does not account for the maintenance of cardiac output associated with delayed cord clamping. Indeed, blood transfusion prior to the onset of ventilation played no role in maintaining higher PBF or triggered the transition to the neonatal circulation. This indicates that the onset of ventilation is the most critical aspect for the maintenance and/or restoration of cardiac output during a successful transition to the neonatal circulation.

## Data Availability Statement

The datasets generated for this study are available on request to the corresponding author.

## Ethics Statement

The animal study was reviewed and approved by Monash University Ethics Committee A.

## Author Contributions

FS, SH, MK, KC, AG, EW, AP, DL, and GP: substantial contributions to the conception or design of the work; or the acquisition, analysis or interpretation of data for the work. FS, SH, MK, AG, EW, AP, and GP: drafting the work or revising it critically for important intellectual content. FS, SH, MK, KC, AG, EW, AP, DL, and GP: provide approval for publication of the content. All authors agree to be accountable for all aspects of the work in ensuring that questions related to the accuracy or integrity of any part of the work are appropriately investigated and resolved.

### Conflict of Interest

The authors declare that the research was conducted in the absence of any commercial or financial relationships that could be construed as a potential conflict of interest.

## Abbreviations

BPM: beats per minute
DCC: delayed cord clamping
FiO_2_: fraction of inspired oxygen
Hb: hemoglobin
HR: heart rate
IVH: intraventricular hemorrhage
LVO: left ventricular output
NIRS: near infrared spectroscopy
PaCO_2_: partial pressure of carbon dioxide
PaO_2_: partial pressure of oxygen
PBF: pulmonary blood flow
P_CA_: carotid arterial pressure
PEEP: peak end expiratory pressure
PIP, peak inspiratory pressure PVR: pulmonary vascular resistance
SctO_2_: cerebral oxygenation
SpO_2_: arterial oxygen saturation as measured by pulse oximetry
SVC: superior vena cava
Vt: tidal volume
VTI: velocity time integral.
